# Epigenome-wide association studies of prenatal maternal mental health and infant epigenetic profiles: a systematic review

**DOI:** 10.1038/s41398-023-02620-1

**Published:** 2023-12-07

**Authors:** Emily Drzymalla, Krista S. Crider, Arick Wang, Gwinn Marta, Muin J. Khoury, Danielle Rasooly

**Affiliations:** 1 Division of Blood Disorders and Public Health Genomics, National Center on Birth Defects and Developmental Disabilities, Centers for Disease Control and Prevention, Atlanta, GA USA; 2 Infant Outcomes Research and Prevention Branch, Division of Birth Defects and Infant Disorders, National Center on Birth Defects and Developmental Disabilities, Centers for Disease Control and Prevention, Atlanta, GA USA; 3 Tanaq Support Services, Atlanta, GA USA

**Keywords:** Genetics, Biomarkers

## Abstract

Prenatal stress and poor maternal mental health are associated with adverse offspring outcomes; however, the biological mechanisms are unknown. Epigenetic modification has linked maternal health with offspring development. Epigenome-wide association studies (EWAS) have examined offspring DNA methylation profiles for association with prenatal maternal mental health to elucidate mechanisms of these complex relationships. The objective of this study is to provide a comprehensive, systematic review of EWASs of infant epigenetic profiles and prenatal maternal anxiety, depression, or depression treatment. We conducted a systematic literature search following PRISMA guidelines for EWAS studies between prenatal maternal mental health and infant epigenetics through May 22, 2023. Of 645 identified articles, 20 fulfilled inclusion criteria. We assessed replication of CpG sites among studies, conducted gene enrichment analysis, and evaluated the articles for quality and risk of bias. We found one repeated CpG site among the maternal depression studies; however, nine pairs of overlapping differentially methylatd regions were reported in at least two maternal depression studies. Gene enrichment analysis found significant pathways for maternal depression but not for any other maternal mental health category. We found evidence that these EWAS present a medium to high risk of bias. Exposure to prenatal maternal depression and anxiety or treatment for such was not consistently associated with epigenetic changes in infants in this systematic review and meta-analysis. Small sample size, potential bias due to exposure misclassification and statistical challenges are critical to address in future efforts to explore epigenetic modification as a potential mechanism by which prenatal exposure to maternal mental health disorders leads to adverse infant outcomes.

## Introduction

Maternal depression and anxiety, both during and after pregnancy, are common and a major public health problem in the United States, affecting over 1 in 10 mothers [1]. Epidemiologic studies have suggested an association between prenatal maternal depression and anxiety with adverse child outcomes such as low birthweight and preterm delivery [2, 3], as well as developmental delays and emotional and behavioral problems [4]. Low birthweight, preterm delivery, and developmental delays have been associated with changes in methylation profiles [5–7]. Epigenetic modifications have been proposed as possible mechanisms that may help explain the association between prenatal stress and adverse child developmental outcomes [8]. The motivation for this approach is that DNA methylation may alter gene expression in ways that influence early-infancy and later-life developmental outcomes.

Epigenetic markers, including DNA methylation, may be responsive to environmental factors throughout life, especially during in utero development [9]. With the exception of imprinted regions, the genome is demethylated prior to implantation with totipotency restored and the appropriate sex and tissue type specificity patterns reestablished throughout development [10–16]. Prenatal smoking [17], body mass index [18], and exposure to certain chemicals [19, 20] have been associated with changes to the infant’s epigenetic profile and the long-term health outcomes of offspring. It has thus been hypothesized that in utero exposure of the fetus to maternal depression may influence infant epigenetic profiles that alter fetal and child health and development. However, exposure to maternal depression is highly related and confounded with many potential variables such as smoking [21], maternal age [22], and maternal socioeconomic status [23]. Alterations in DNA methylation patterns in response to maternal depression may be adaptive changes that help an infant to anticipate a stressful or scarce environment; alternatively, they could be induced by pathological changes associated with medications or increased oxidative stress, or perhaps simply reflect different underlying sequence variation.

Epigenome-wide associations studies (EWAS) investigate associations between a phenotype (e.g. maternal mental health) and epigenetic variants in various tissues across the genome spanning 27,000 to a million or more CpG methylation sites [24]. While most studies of prenatal stress and infant epigenetic outcomes have focused on candidate gene methylation sites, epigenome-wide studies take an agnostic approach to identifying novel associations. Determining the influence of prenatal exposures on offspring epigenomic patterns requires complex study design and careful consideration of potential confounding and effect modification because the genetic, epigenetic, and environmental exposures over time of two linked but independent individuals must be considered. Although several reviews have attempted to synthesize related research [25–27], to our knowledge, ours is the first comprehensive, systematic review of EWAS of prenatal mental health and infant epigenetic profiles.

We performed a systematic review and critical assessment of EWAS studies on maternal mental health during pregnancy and the epigenetic profile of the offspring to assess whether depression, depression treatment, or anxiety in pregnancy women may influence offspring epigenetic profiles, compared with the epigenetic profiles in offspring born to mothers without depression or anxiety during pregnancy. We examined each study’s design, statistical analyses, and reporting, as well as its methodological quality and risk of bias. We assessed replication of CpG findings among studies and conducted gene enrichment analysis. Our findings underscore the importance of replication of research and study design in this area; more studies are needed to clarify the associations between maternal mental state and offspring epigenetic changes, which may influence offspring early-infancy and later-life health and developmental outcomes.

## Methods

### Literature search

We conducted our systematic review in accordance with the Preferred Reporting Items for Systematic Reviews and Meta-Analyses (PRISMA) guidelines [28] and registered it prospectively in PROSPERO (registration ID number: CRD42022335595). We conducted a review of EWAS to assess the association between mothers with vs mothers without either maternal depression, maternal anxiety, or depression treatment during pregnancy and their offspring’s epigenetic profile to explore the quality of these studies and compare their findings. The exposures were maternal depression, maternal anxiety, or depression treatment during pregnancy. The comparison groups were mothers who did not experience maternal depression, maternal anxiety, or depression treatment during pregnancy. The outcomes were epigenetic profiles of the offspring. Epigenome wide association studies were eligible for inclusion if they 1) specified the epigenetic profile of the offspring as the outcome, 2) measured exposures occurring during pregnancy, and 3) were published in the English language. Studies were excluded if they 1) analyzed only candidate genes, or 2) were published only as conference papers or abstracts. The search was conducted on May 22, 2023, on PubMed, Scopus, and Embase. The following search terms were applied: “epigenome wide association study”, “DNA methylation”, “pregnancy”, “prenatal”, “depression”, “anxiety”, and “psychiatric” (detailed search strategy is available in the Supplement).

### Data extraction and quality assessment

The results of the search were exported to Excel and two reviewers (ED, KSC) conducted title abstract screening full text of articles that passed screening. Risk of bias was assessed by two reviewers (ED and DR) through the Risk of Bias in Non-randomized Studies of Interventions (ROBINS-I) tool [29] and the quality of reporting was assessed using the Strengthening the Reporting of Observational Studies in Epidemiology (STROBE) guidelines [30]. From each study, we extracted data pertaining to sample size, recruitment time period, time of enrollment, country, recruitment location, ethnicity/race, age, study design, analytic method, methylation chip, tissue sample type, cell type correction, covariates, covariate data collection methods, ancestral markers, genetic interactions, genome-wide associations, CpG sites excluded from analysis, *P*-values for significance, replication/validation analyses, exposures, exposure measurements, timing of exposure measurement, main CpG findings, main DMR findings, and relevant genes. The significant or top CpG sites and DMRs were compared within each exposure category to find any replicated CpG sites and DMRs or overlapping DMRs. Gene enrichment of the gene ontology (GO) categories of the significant and top ranked CpG sites and DMRs from the studies was conducted using the gometh function of the missMethyl R package [31] (version 1.31.0). Gometh tests GO enrichments for inputted CpG sites by empirically calculating the probability of differential methylation as a function of the number of CpGs, which accounts for biases in the number of probes per gene on the array and for CpGs that are annotated to multiple genes [31].

**Fig. 1:**
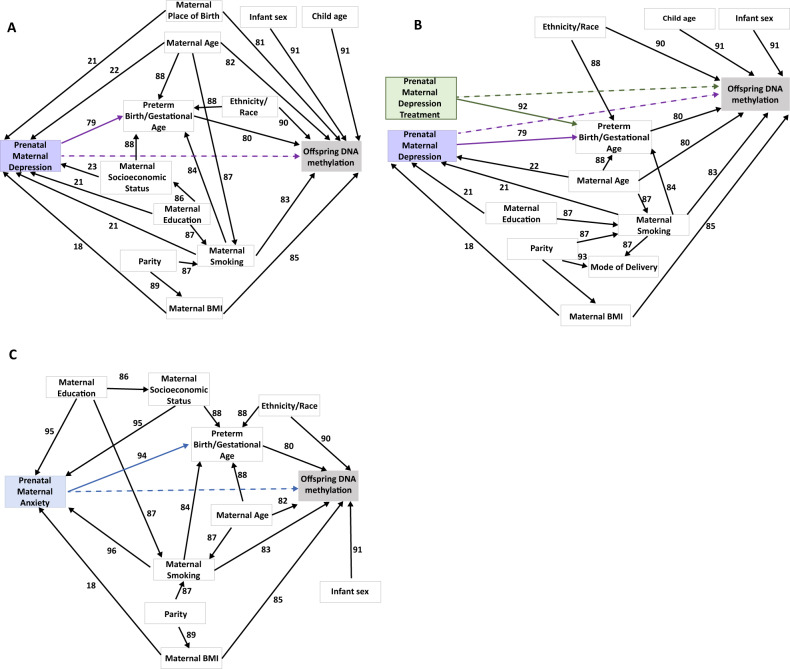
Directed acyclic graph for prenatal maternal health exposures and offspring DNA methylation profile. **A** Directed acyclic graph for prenatal maternal depression and offspring DNA methylation profile. Many factors have been associated with prenatal maternal depression, offspring DNA methylation, or both resulting in a complex network between prenatal maternal depression and offspring DNA methylation. The arrows represent associations between two factors in which one factor influences the other. The hyphened arrows refer to a theoretical association. The purple arrows refer to associations where prenatal maternal depression influences another factor, and the black arrows refer to associations between the covariates and other factors. These covariates came from common covariates used in the studies for prenatal maternal depression and offspring DNA methylation in this review. Prenatal maternal depression can be connected to or influence offspring DNA methylation through multiple pathways. A main pathway through which prenatal maternal depression influences offspring methylation in this DAG is through preterm birth/gestational age [79, 80]. Multiple factors including maternal place of birth [21, 81], maternal age [22, 82], maternal smoking [21, 83, 84], maternal BMI [18, 85], maternal education [21, 86, 87], maternal SES [23, 88], parity [87, 89], and race/ethnicity [88, 90] influence both prenatal maternal depression and offspring DNA methylation. Though not “directly” connected in this DAG, offspring DNA methylation is influenced by maternal education through maternal smoking [87], by maternal socioeconomic status through preterm birth/gestational age [88], and by parity through maternal smoking [87] and maternal BMI [89]. Infant sex [91] and child age [91] also influence the offspring’s DNA methylation profile. **B** Directed acyclic graph for prenatal maternal depression treatment and offspring DNA methylation profile. Many factors have been associated with prenatal maternal depression treatment, offspring DNA methylation, or both resulting in a complex network between prenatal maternal depression and offspring DNA methylation. The arrows represent associations between two factors in which one factor influences the other. The hyphened arrows refer to a theoretical association. The arrows represent associations between two factors in which one factor influences the other. The green arrows refer to associations where prenatal maternal depression treatment influences another factor, the purple arrows refer to association where prenatal maternal depression influences another factor, and the black arrows refer to associations between the covariates and another factors. These covariates came from common covariates used in the studies for prenatal maternal depression treatment and offspring DNA methylation in this review. Like prenatal maternal depression, prenatal maternal depression treatment influences offspring DNA methylation through preterm birth/gestational age [92]. As with the previous DAG in **A**, maternal age [22, 82], maternal smoking [21, 83], maternal BMI [18, 85], and parity [87, 89, 93] maternal education [21, 81] influence both prenatal maternal depression and offspring DNA methylation. As a result, these factors also influence prenatal maternal depression treatment through prenatal maternal depression. Infant sex [91] and child age [91] also influence the offspring’s DNA methylation profile. **C** Directed acyclic graph for prenatal maternal anxiety and offspring DNA methylation profile. Many factors have been associated with prenatal maternal anxiety, offspring DNA methylation, or both. The arrows represent associations between two factors in which one factor influences the other. The blue arrows refer to associations where prenatal maternal anxiety influences another factors and the black arrows refer to associations between the covariates and another factors. The hyphened arrow refers to a theoretical association. These covariates came from common covariates used in the studies for prenatal maternal anxiety and offspring DNA methylation in this review. Prenatal maternal anxiety is connected to or influences offspring DNA methylation through multiple pathways. An important route in which prenatal maternal anxiety influences offspring DNA methylation is through preterm birth/gestational age [94]. Similarly, to **A** and **B**, except with prenatal maternal anxiety instead of prenatal maternal depression, maternal education [86, 95], maternal socioeconomic status [88, 95], maternal smoking [83, 96], maternal age [82, 87], maternal BMI [18, 85], and parity [87, 89] influence both prenatal maternal anxiety and offspring DNA methylation. Infant sex [91] also influences the offspring’s DNA methylation profile.

We conducted separate analyses for three specific associations: prenatal maternal depression and offspring DNA methylation profile (directed acyclic graph Fig. 1A), prenatal maternal depression treatment and offspring DNA methylation profile (directed acyclic graph Fig. 1B), and prenatal maternal anxiety and offspring DNA methylation profile (directed acyclic graph Fig. 1C). Due to the direct relationship between depression and depression treatment shown in Fig. 1B and the effect medications may have on DNA methylation, depression treatment was examined as its own category. Anxiety treatment was not examined due to the lack of articles on anxiety treatment in the search results.

## Results

### Included studies

**Fig. 2:**
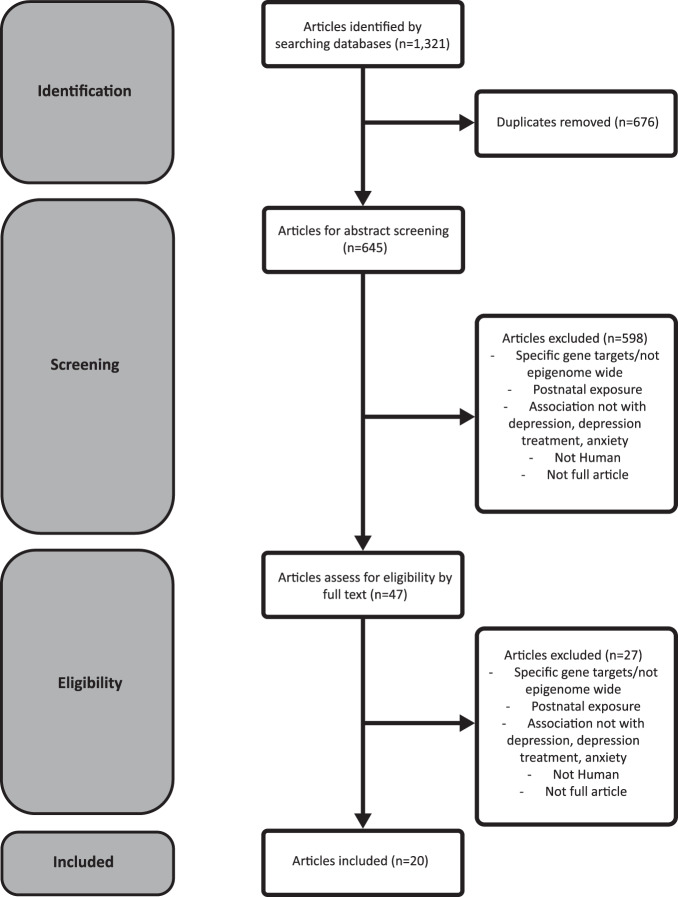
Preferred Reporting Items for Systematic Reviews and Meta-Analyses flow diagram. Preferred Reporting Items for Systematic Reviews and Meta-Analyses (PRISMA) flow diagram for epigenome wide association studies about maternal mental health and infant epigenetic profile curation.

We identified 1321 articles from our PubMed, Scopus, Embase search. Of these, 676 were excluded as duplicates leaving 645 for abstract screening. After removing 598 during abstract screening, we performed full text screening on the remaining 47 articles and removed an additional 27 for one of the following reasons: non-human study population, candidate genes only, postnatal exposure, not being a full article, or exposure other than maternal depression, maternal depression treatment, or maternal anxiety, The remaining 20 articles were included our analysis (Fig. 2). Several articles described analyses of more than one exposure, resulting in 16 analyses [32–47] of maternal depression, 8 analyses [32, 35, 38, 39, 44, 45, 47, 48] of maternal depression treatment, and 9 analyses [27, 32, 33, 35, 38, 41, 47, 49, 50] of maternal anxiety (Table S1). Based on STROBE and ROBINS-I criteria, we found that 13 analyses had a moderate risk of bias and 7 had a severe risk of bias (Tables S2 and S3).

### Depression during pregnancy

Of the 16 studies that analyzed associations between maternal depression during pregnancy and the infant’s epigenetic profile, we found the risk of bias to be moderate in 12 studies and severe in 4 studies. Main factors increasing the risk of bias were not accounting for all important confounders and not accounting for selection bias. Covariates and confounders adjusted for in more than one of the included studies are shown in Fig. 1A. No statistically significant CpG site was reported from more than one study. However, one CpG site, cg25157095, was reported as a top ranked CpG site in two studies. Nine pairs of overlapping DMRs were found in at least two maternal depression studies (Table 1). The gene enrichment analysis had 62 significant pathways for study significant DMRs and 65 signficant pathways for top ranked DMRs (Table 2). The gene enrichment analysis did not produce any significant results for the CpG sites. The top ten pathways for the CpG sites are presented in Table S4.

**Table 1:** Overlapping differential methylated regions and repeated CpG site for maternal depression.

Overlapping DMRs	Studies	Maternal depression measurement	Closest gene	*P*-value
chr8:70378380-70378994	Drzymalla et al.	BDI-II (threshold 20)	*SULF1*	3.28 × 10^−4^
chr8:70378380-70378995	Viuff et al.	EPDS (threshold 12)		8.0 × 10^−^^5^
chr7:27183643-27184853	Drzymalla et al.	BDI-II (threshold 14)	*HOXA*	1.79 × 10^−^^3^
chr7:27183133-27184522	Viuff et al.	EPDS (threshold 12)		8.4 × 10^−^^10^
chr1:62660188-62660861	Drzymalla et al.	EPDS (threshold 13)	*L1TD1*	4.67 × 10^−^^7^
chr1:62660038-62661010	Robakis et al.	EPDS (continuous)		2.10 × 10^−^^3^
chr8: 143859669-143859991	Viuff et al.	EPDS (threshold 12)	*LYNX1*	1.2 × 10^−^^5^
chr8: 143859369-143860092	Robakis et al.	EPDS (continuous)		3.66 × 10^−^^2^
chr6: 151125848-151125886	Viuff et al.	EPDS (threshold 12)	*PLEKHG1*	3.0 × 10^−^^5^
chr6: 151125729-151125904	Robakis et al.	EPDS (continuous)		1.55 × 10^−^^2^
chr8: 70602451-70602610	Viuff et al.	EPDS (threshold 12)	*SLCO5A1*	2.0 × 10^−^^2^
chr8: 70602401-70602611	Robakis et al.	EPDS (continuous)		2.25 × 10^−^^2^
chr1: 242018546-242018546	Stonawski et al.	EPDS (threshold 10)	*EXO1*	0.59*
chr1: 242017974-242019110	Robakis et al.	EPDS (continuous)		3.72 × 10^−^^2^
chr7: 158649758-158649758	Stonawski et al.	EPDS (threshold 10)	*WDR60*	1.00*
chr7: 158649387-158649760	Robakis et al.	EPDS (continuous)		3.80 × 10^−^^2^
chr11: 85195094-85195206	Viuff et al.	EPDS (threshold 12)	*DLG2*	3.1 × 10^−^^2^
chr11: 85195119-85195288	Robakis et al.	EPDS (continuous)		9.69 × 10^−^^3^

CpG site	Studies	Maternal Depression Measurement	Closest gene	*P*-value
cg25157095	Stonawski et al.	EPDS (threshold 10)	*RIPK4*	4.14 × 10^−^^5^
cg25157095	Bleker et al.	BDI-II (threshold 29)		2.84 × 10^−^^4^

* FDR *p*-value.

**Table 2:** Significant pathways for DMRs.

Pathway	Number of genes in term pathway	Number of differentially methylated genes	*p*-value	FDR
**Study significant DMRs**				
Double-stranded DNA binding	1649	565	4.13E−06	0.0069
External encapsulating structure	572	222	4.20E−06	0.0069
Outflow tract morphogenesis	80	46	4.70E−06	0.0071
Collagen-containing extracellular matrix	433	173	5.04E−06	0.0071
Sequence-specific double-stranded DNA binding	1552	534	5.47E−06	0.0071
RNA polymerase II cis-regulatory region sequence-specific DNA binding	1186	418	5.56E−06	0.0071
Nervous system development	2483	896	7.97E−06	0.0090
Cardiac chamber development	167	83	8.23E−06	0.0090
Transcription regulator activity	1888	645	8.28E−06	0.0090
Intracellular anatomical structure	15038	4466	8.71E−06	0.0091
Animal organ development	3567	1202	1.14E−05	0.0114
Cardiac chamber morphogenesis	126	65	1.21E-05	0.0116
Cellular developmental process	4298	1428	1.34E−05	0.0123
Developmental process	6471	2088	1.65E−05	0.0146
Sequence-specific DNA binding	1653	562	1.75E−05	0.0147
DNA binding	2456	806	1.85E−05	0.0147
Cellular component organization	6221	2029	1.86E−05	0.0147
Cell differentiation	4271	1417	2.06E−05	0.0158
Intracellular membrane-bounded organelle	12329	3672	2.19E−05	0.0162
Intracellular organelle	13404	3992	2.27E−05	0.0163
Cation channel activity	343	146	2.55E−05	0.0178
Neurogenesis	1667	622	2.68E−05	0.0181
Binding	16179	4769	2.93E−05	0.0192
Circulatory system development	1207	434	3.16E−05	0.0201
Cell development	2151	773	3.45E−05	0.0214
Regulation of primary metabolic process	5846	1835	3.71E−05	0.0219
Embryo development ending in birth or egg hatching	675	264	3.72E−05	0.0219
Tissue development	1982	692	4.28E−05	0.0237
Plasma membrane bounded cell projection organization	1506	563	4.36E−05	0.0237
System development	4429	1479	4.37E−05	0.0237
Chordate embryonic development	653	256	4.44E−05	0.0237
Regulation of nitrogen compound metabolic process	5684	1785	5.15E−05	0.0269
Macromolecule biosynthetic process	4702	1483	5.73E−05	0.0286
Heart morphogenesis	258	113	5.74E−05	0.0286
RNA biosynthetic process	3497	1135	5.94E−05	0.0289
Organic cyclic compound binding	6189	1906	6.06E−05	0.0289
Multicellular organism development	4896	1622	6.43E−05	0.0301
Bone morphogenesis	96	51	6.66E−05	0.0305
Regulation of cellular metabolic process	5630	1773	6.89E−05	0.0308
Transcription by RNA polymerase II	2621	867	7.10E−05	0.0308
Nucleic acid-templated transcription	3480	1129	7.10E−05	0.0308
Regulation of nucleobase-containing compound metabolic process	3990	1284	7.50E−05	0.0319
Neuronal cell body	496	201	7.71E−05	0.0321
DNA-templated transcription	3478	1128	7.89E−05	0.0321
Regulation of RNA biosynthetic process	3375	1097	7.97E−05	0.0321
Growth	918	344	8.28E−05	0.0328
Nucleic acid binding	4190	1268	8.54E−05	0.0332
Membrane-bounded organelle	13489	3979	9.05E−05	0.0338
Embryo development	1128	419	9.05E−05	0.0338
Regulation of transcription by RNA polymerase II	2536	840	9.13E−05	0.0338
Gated channel activity	340	143	9.51E−05	0.0346
Regulation of nucleic acid-templated transcription	3366	1093	9.99E−05	0.0358
Ion gated channel activity	44	27	1.05E−04	0.0369
Regulation of DNA-templated transcription	3364	1092	1.06E−04	0.0369
Positive regulation of proteolysis	372	147	1.10E−04	0.0375
Regulation of developmental process	2566	874	1.13E−04	0.0381
Organelle	14302	4218	1.31E−04	0.0436
Neuron development	1111	427	1.38E−04	0.0453
Cell morphogenesis involved in differentiation	724	295	1.47E−04	0.0467
Heart development	605	236	1.47E−04	0.0467
Regulation of RNA metabolic process	3672	1185	1.51E−04	0.0475
**Top DMRs**				
Cis-regulatory region sequence-specific DNA binding	1206	429	2.73E−06	0.0067
Cellular component organization or biogenesis	6426	2103	2.97E−06	0.0067
Cardiac ventricle development	126	67	3.20E−06	0.0067
Double-stranded DNA binding	1649	567	3.97E−06	0.0076
Extracellular matrix	571	222	4.70E−06	0.0077
Outflow tract morphogenesis	80	46	5.18E−06	0.0077
Sequence-specific double-stranded DNA binding	1552	536	5.33E−06	0.0077
External encapsulating structure	572	222	5.40E−06	0.0077
RNA polymerase II cis-regulatory region sequence-specific DNA binding	1186	419	6.12E−06	0.0080
Collagen-containing extracellular matrix	433	173	6.26E−06	0.0080
Transcription regulator activity	1888	647	8.46E−06	0.0101
Cardiac chamber development	167	83	9.44E−06	0.0101
Nervous system development	2483	898	9.59E−06	0.0101
Intracellular anatomical structure	15038	4481	9.69E−06	0.0101
DNA binding	2456	811	1.13E−05	0.0112
Sequence-specific DNA binding	1653	565	1.31E−05	0.0120
Animal organ development	3567	1205	1.33E−05	0.0120
Cardiac chamber morphogenesis	126	65	1.36E−05	0.0120
Cellular developmental process	4298	1432	1.47E−05	0.0125
Cellular component organization	6221	2036	1.78E−05	0.0146
Developmental process	6471	2094	1.88E−05	0.0148
Cell differentiation	4271	1421	2.27E−05	0.0174
Embryo development ending in birth or egg hatching	675	266	2.36E−05	0.0175
Intracellular membrane-bounded organelle	12329	3684	2.44E−05	0.0175
Intracellular organelle	13404	4005	2.58E−05	0.0179
Chordate embryonic development	653	258	2.83E−05	0.0189
Binding	16179	4786	2.88E−05	0.0189
Cation channel activity	343	146	3.07E−05	0.0195
Neurogenesis	1667	623	3.27E−05	0.0199
Regulation of primary metabolic process	5846	1842	3.30E−05	0.0199
Cell development	2151	775	3.71E−05	0.0218
Neuronal cell body	496	203	4.42E−05	0.0241
Circulatory system development	1207	434	4.48E−05	0.0241
Regulation of nitrogen compound metabolic process	5684	1792	4.49E−05	0.0241
Regulation of cellular metabolic process	5630	1782	4.52E−05	0.0241
Transcription by RNA polymerase II	2621	872	4.78E−05	0.0247
RNA biosynthetic process	3497	1140	4.85E−05	0.0247
Macromolecule biosynthetic process	4702	1489	4.94E−05	0.0247
Plasma membrane bounded cell projection organization	1506	564	5.08E−05	0.0248
Tissue development	1982	693	5.43E−05	0.0260
Organic cyclic compound binding	6189	1913	5.64E−05	0.0260
System development	4429	1482	5.68E−05	0.0260
Nucleic acid-templated transcription	3480	1134	5.79E−05	0.0260
Growth	918	346	6.23E−05	0.0275
DNA-templated transcription	3478	1133	6.42E−05	0.0275
Nucleic acid binding	4190	1274	6.65E−05	0.0275
Heart morphogenesis	258	113	6.69E−05	0.0275
Regulation of nucleobase-containing compound metabolic process	3990	1289	6.70E−05	0.0275
Regulation of transcription by RNA polymerase II	2536	844	7.29E−05	0.0287
Bone morphogenesis	96	51	7.30E−05	0.0287
Embryo development	1128	421	7.45E−05	0.0287
Regulation of RNA biosynthetic process	3375	1101	7.50E−05	0.0287
Multicellular organism development	4896	1626	7.67E−05	0.0288
Membrane-bounded organelle	13489	3993	8.99E−05	0.0332
Regulation of nucleic acid-templated transcription	3366	1097	9.42E−05	0.0343
Regulation of DNA-templated transcription	3364	1096	1.00E−04	0.0359
Ion gated channel activity	44	27	1.11E−04	0.0392
Gated channel activity	340	143	1.13E−04	0.0392
Organelle	14302	4233	1.28E−04	0.0434
Regulation of developmental process	2566	876	1.29E−04	0.0434
Positive regulation of proteolysis	372	147	1.31E−04	0.0435
Regulation of biological quality	3744	1232	1.46E−04	0.0475
Neuron development	1111	428	1.47E−04	0.0475
Regulation of RNA metabolic process	3672	1189	1.50E−04	0.0477

### Depression treatment during pregnancy

Eight studies analyzed associations of maternal depression treatment during pregnancy with the infant’s epigenetic profile. We found the risk of bias to be moderate in 7 of these studies and severe in 1 study. Main factors increasing the risk of bias in these studies were not accounting for all important confounders, not accounting for selection bias, and missing data. Covariates and confounders adjusted for in more than one of the included studies are shown in Fig. 1B. No statistically significant or top-ranked CpG sites were reported from more than one study. Many studies either did not perform a DMR analysis or did not provide ranked results. Only one DMR, chr12: 56325797–56325867, was reported as a top ranked DMR. The gene enrichment analysis did not produce any significant results for the CpG sites. The top ten pathways are presented in Table S5.

### Anxiety during pregnancy

Of the 9 studies that analyzed the association between maternal anxiety during pregnancy and the infant’s epigenetic profile, 7 had a moderate risk of bias while 2 had a severe risk of bias. Main factors increasing the risk of bias in these studies were not accounting for all important confounders, not accounting for selection bias, and missing data. Covariates and confounders adjusted for in more than one of the included studies are shown in Fig. 1C. No statistically significant or top-ranked CpG sites were reported from more than one study. The studies shared no overlapping DMRs. The gene enrichment analysis did not produce any significant results for the CpG sites or the DMRs. The top ten pathways are presented in Table S6.

## Discussion

To our knowledge, the present work is the first systematic review of epigenome-wide association studies of prenatal maternal mental health and infant epigenetic profiles. We identified 20 EWAS studies which together reported 803 CpG sites and 19,440 DMRs in infants associated with maternal anxiety, depression, or depression treatment. Among the studies within any maternal mental health category, there was only one top ranked CpG site, cg25157095, reported in more than one study. Only nine overlapping DMRs were reported in at least two studies on maternal depression. We identified significant pathways only for maternal depression DMRs in the gene enrichment analysis. The main limitations of the studies we identified were small sample sizes, concerns about exposure misclassification and suboptimal statistical analyses that increased the risk of bias.

We found that no replicated single significant CpG sites were reported from more than one study in any of the three categories (maternal depression, maternal depression treatment, and maternal anxiety). However, one CpG site, cg25157095 was found among the top non-significant CpG sites for maternal depression in two studies [40, 47]. This CpG site is in an intron for the *RIPK4* gene which has been implicated in keratinocyte differentiation, modulation of the actin cytoskeleton, and restricting intercellular adhesion [51]. Three pairs of overlapping DMRs were reported in two of 15 studies of maternal depression during pregnancy. One pair of overlapping DMRs, chr8:70378380-70378994 and chr8:70378380-70378995, is in the *SULF1* gene. The *SULF1* gene is involved in the regulation of multiple cellular pathways for editing heparan sulfate chains [52]. This gene has been associated with nervous system development in studies of SULF1 deficient mice, providing evidence that a non-functioning *SULF1* gene is associated with impaired neurite growth and impaired long-term potentiation [53, 54], a form of synaptic plasticity [55]. Single nucleotide polymorphisms (SNPs) in this gene have been associated with cancer risk [56]. Another pair of overlapping DMRs, chr8: 143859669-143859991 and chr8: 143859369-143860092, is closest to the *LYNX1* gene. Evidence has been provided through mouse models for the possible role of this gene in synaptic plasticity [57].

The chr11: 85195094-85195206 and chr11: 85195119-85195288 pair of overlapping DMRs are in the *DLG2* gene. This gene encodes for the postsynaptic density 93 protein which has been thought to have roles in synaptic stability and regulation [58, 59]. Genetic variations in *DLG2* have been associated with schizophrenia [60], attention deficit hyperactivity disorder [61], and bipolar disorder [62]. Another pair of overlapping DMRs were chr7:27183643-27184853 and chr7:27183133-27184522, which were significantly associated in opposite directions in the two studies. This DMR is located in *HOXA-5* in the *HOXA* gene cluster, which is important in human development [63]. Hypermethylation in this gene has also been associated with various cancers [64] and hypermethylation in this region of the gene has also been associated with Alzheimer’s disease [65]. Another pair of overlapping DMRs, chr1:62660188-62660861 and chr1:62660038-62661010, span the *L1TD1* gene. The *L1TD1* gene has been connected to pluripotency maintence [66]. Higher expression of *L1TD1* has also been associated with longer disease free colon cancer survival [67] and *L1TD1* has been found to have higher levels of methylation in non-small cell lung cancer tissue [68]. The chr6: 151125848-151125886 and chr6: 151125729-151125904 pair of DMRs are within the *PLEKHG1* gene. A SNP within this gene has been associate with white matter hyperintensities and ischemic stroke [69].

Continued research in this area is needed to determine if changes in these DMR regions result in functional changes that may correspond to the adverse outcomes seen in the epidemiological literature [2–4]. The overall lack of replication across studies highlights the difficulty of interpreting these types of analyses.EWAS studies are limited by the types of CpG sites included and may systematically overlook critical regions [70, 71]. These types of studies also limited by available tissue type, typically blood or saliva. It is well established that DNA methylation patterns are tissue specific and the lack of data on critical regions such as the brain is problematic [11, 70, 72]. Poor correlation of DNA methylation results measured using different Illumina platforms (e.g., the EPIC, 450k, and 27k arrays) presents problems for replication [73]. Underlying sequence variation is also of concern in EWAS studies as methylation can be a direct result of the underlying genetic sequence. Correction for population stratification and genetic variation/interaction will be critical in future studies.

Methods for determining and classifying maternal depression and maternal anxiety during pregnancy varied among our included studies, as did measurements of medication intake and dosage. The studies we considered to have a severe risk of bias all lacked appropriate adjustment for confounding and consideration of selection bias.

Maternal depression is a critical public health problem that is both under-treated and under-diagnosed, especially in minority and underserved populations. Untreated prenatal depression has been associated with detrimental health outcomes for both the pregnant woman and the baby, including an elevated risk for postpartum depression in the mother and increased infant risk for preterm birth and low birth weight [74]. Antenatal maternal anxiety and stress can impact the psychological and intellectual development of the infant [75], with some studies suggesting increased risk for emotional and cognitive problems, attentional deficit, and language delay. Prenatal anxiety and depression are also associated with increased risk for suicidality in mothers [76], with the greatest increases seen among Non-Hispanic Black, low-income, and younger individuals. Maternal mental health is tied to racial and ethnic disparities, with a higher overall prevalence of maternal depression among non-Hispanic Blacks and Hispanics compared to non-Hispanic whites [77]. Emerging evidence indicates that the COVID-19 pandemic increased the prevalence of mental health issues during pregnancy, with a meta-analysis of 37 studies suggesting that more than one in four pregnant women experienced prenatal depression and one in three experienced clinically significant anxiety [78]. Early screening and treatment for women at risk for maternal depression and anxiety may help prevent long-term adverse outcomes on maternal and infant well-being.

The EWAS studies included in this systematic review explored associations of prenatal anxiety or depression with DNA methylation patterns in offspring. Among the included studies, there was a lack of replication for a majority of the studies’ findings. However, a limitation of this study includes the potential to miss relevant articles in the initial search. Further studies of larger sample sizes are needed to identify and replicate findings and further investigate the role of maternal mental health on infant epigenetic profiles as well as take greater steps to control for confounding and address selection bias.

### Supplementary information

Table S1

Table S2

Table S3

Table S4

Table S5

Table S6
